# Comparative effectiveness of biorational pesticides for management of *Phenacoccus solenopsis* Tinsley and *Paracoccus marginatus* Williams & Granara de Willink in *Gymnema sylvestre* (Retz.) R.Br. ex Sm

**DOI:** 10.1016/j.heliyon.2023.e23648

**Published:** 2023-12-13

**Authors:** Shivakumara Kadanakuppe Thammayya, Keerthi Manikyanahalli Chandrashekara, Akula Chinapolaiah, Ramya Ramesan Syamala, Shivakumar Kadukothanahalli Veerabhadraiah, Bhemanna Somanna Gotyal, Manjunatha Channappa, Ryan Casini, Ihab Mohamed Moussa, Hosam O. Elansary, Ahmed M. El-Sabrout

**Affiliations:** a Division of Genomic Resources, ICAR-National Bureau of Agricultural Insect Resources, Bengaluru, Karnataka, 560024, India; b ICAR-Directorate of Medicinal and Aromatic Plant Research, Anand, Gujarat, 387310, India; c Division of Crop Protection, ICAR-Indian Institute of Horticultural Research, Bengaluru, Karnataka, 560089, India; d ICAR-Directorate of Floricultural Research, Pune, Maharashtra, 411036, India; e School of Public Health, University of California, Berkeley, 2121 Berkeley Way, Berkeley, CA, 94704, USA; f Department of Botany and Microbiology, College of Science, King Saud University, P.O. Box-2455, Riyadh, 11451, Saudi Arabia; g Plant Production Department, College of Food and Agriculture Sciences, King Saud University, Riyadh, 11451, Saudi Arabia; h Department of Applied Entomology and Zoology, Faculty of Agriculture (EL-Shatby), Alexandria University, Alexandria, 21545, Egypt

**Keywords:** *Gymnema sylvestre*, *Phenacoccus solenopsis*, *Paracoccus marginatus*, Azadirachtin, Entomopathogenic fungi, Mortality, Lethal effect

## Abstract

The cotton mealybug, *Phenacoccus solenopsis* Tinsley and papaya mealybug, *Paracoccus marginatus* Williams and Granara de Willink (Hemiptera: Pseudococcidae) are becoming major threats to the production of *Gymnema sylvestre* R. Br. (Asclepiadaceae) in India. Management mainly depends on chemical insecticides which cause a serious problem of pesticide residue and insecticide resistance. The use of biorational insecticides such as biopesticides, botanicals, insect growth regulators, and microbial insecticides is important components of an Integrated Pest Management (IPM) program for successful management. We evaluated the bio-efficacy of twelve biorational insecticides, including entomopathogenic fungi (EPF), using the leaf spray method in laboratory conditions at 25 ± 1 °C, 70 % ± 5 % RH. The results revealed that the highest percent mortality was recorded by acetamiprid 20 % SP (100.00 %), followed by azadirachtin (98.27 %), *Lecanicillium muscarium* (2 × 10^9^ spores/mL) (85.70 %) and *Ocimum sanctum* leaf extract (76.87 %) at 120 h after treatment (HAT) in *P. solenopsis*. In *P. marginatus*, 100.00 %, 96.39 % and 85.67 % and 74.90 % mortalities were achieved by acetamiprid 20 % SP, azadirachtin, *L. muscarium* (2 × 10^9^ spores/mL) and *O. sanctum* leaf extract, respectively, at 120 HAT during the first spray. Various biorational insecticides showed a more or less similar trend of percent mortality in both species during the second spray. In both species, the lowest percent mortality was recorded by *Andrographis paniculata* leaf extract (46.29, 44.54) and (41.03, 46.39) at 120 Hours after treatment in the first and second spray, respectively. It was concluded that all the prescribed treatments are more effective than the control. Overall, azadirachtin recorded the highest percent mortality after acetamiprid and had the shortest LT_50_ (12.52 h) and (13.87 h) values in *P. solenopsis* and *P. marginatus*, respectively. Our study emphasizes that biopesticides like Azadirachtin 1 % EC (10000 ppm), *L. muscarium* (2 × 10^9^ spores/mL) (5 mL/L) and *O. sanctum* leaf extract (5 %) may be recommended as alternatives to synthetic insecticides. Botanicals and EPF would be the most effective approach for sustainable integrated management of *P. solenopsis* and *P. marginatus* in the *G. sylvestre* ecosystem.

## 1 Introduction

India has a rich heritage of medicinal and aromatic plants used for preventive and therapeutic medicine. Indian Traditional System of Medicine has been a fundamental part of delivering healthcare to human civilization since its inception. Indian traditional medicine, including Ayurveda, Yoga, Unani, Siddha, and homoeopathy (AYUSH), is unique in the world [1]. Even today, the traditional herbalists are known to practice the herbal medical system in rural regions, utilizing about 2500 herbs to treat common illnesses [2]. India ranks second in terms of exports and offers the highest quality and quantity of medicinal plants. It is one of the world's 12 mega biodiversity hotspots, with 16 agroclimatic zones, and a variety of over 45,000 plant species, 7000 of which are known to be medicinal plants [3]. *Gymnema sylvestre* (Retz.) R.Br. ex Sm. (Apocynaceae) is an important antidiabetic, industrial, and medicinal plant indigenous to the Western Ghats of India and widely distributed in northern and western parts of the country [4], commonly used to treat Diabetes mellitus [5]. *Gymnema sylvestre* is popular known as a sugar destroyer due to its ability to reduce excess body sugar and, is commonly called ‘madhunashini’ [6]. *Gymnema sylvestre* is world's second-best-selling medicinal herb, with a huge demand in domestic and international markets [7]. The major bioactive components groups found in *Gymnema* consist of oleanane-type triterpenoid saponins known as gymnemic acids [4,8]. Gymnemic acid is a main phytoconstituent found in different plants. It has pharmacological properties like suppressing taste sensitivity to sweetness, lowering the plasma glucose level and inhibiting intestinal glucose absorption [9]. In developing countries such as India, only a few medicinal plants are used to control plasma glucose levels with minimal side effects, among which *G. sylvestre* has a huge demand as an antidiabetic medicinal plant [9].

Considering the importance and growing demand, the plant deserves serious attention from researchers in many ways. Despite being grown in field conditions as well as in the wild, lack of agro techniques has made the species vulnerable to multiple stresses in India [10]. The production and quality of the leaf are adversely affected by a complex of biotic and abiotic factors. Among them, insects are the most important limiting factor in field conditions. Two invasive mealybug species, *viz*. papaya mealybug, *Paracoccus marginatus* Williams and Granara de Willink and cotton mealybug, *Phenacoccus solenopsis* Tinsley (Hemiptera: Pseudococcidae), are persistent pests causing significant damage, with the severity of damage ranging from 4 to 91.75 % in India [11,12]. The infestation was observed on all the above-ground parts, significantly affecting new growth flushes. In addition to direct feeding damage, the mealybugs also produce a copious quantity of honeydew, leading to the development of sooty mould, which interferes with photosynthetic capacity and affects the medicinal property of the leaf [12].

Mealybugs are highly polyphagous and destructive pests of various agriculture and horticultural crops distributed globally. The *P. solenopsis* and *P*. *marginatus* are highly invasive, expanding their host range and causing significant yield losses to *G. sylvestre* in India, especially during the summer. The mealybug complex usually attacks all above-ground plant parts of *Gymnema*, *viz*. shoots, leaves, stem and fruits [12]. To date, *P. solenopsis* has been documented in 219 plants, including 70 families worldwide and in India, 194 host plants, including 108 weed hosts with 50 families were recorded [13]. *P. marginatus* was recorded from 136 plants belonging to 49 plant families [14]. Mealybugs are difficult to control with synthetic insecticides due to their cryptic feeding habit, waxy body nature, formation of dense colonies and overlapping generations. Therefore, there is a need for evaluating alternative self-perpetuating natural agents like entomopathogens [15].

Timely interventions, *viz*. cultural practices, biocontrol agents, and chemical insecticides are essential to avoid significant damage to the crop [16]. Farmers currently rely on synthetic insecticides to control mealybugs, which not only raise production costs, but also pose severe risks to operators, consumers, natural enemies and the environment [17,18]. Using synthetic insecticides increases the insecticidal load on the crop and reduces the crop quality by altering the plant's biochemical constituents. Hence, considering the adverse effect of synthetic insecticides, there is a huge scope for the use of biological insecticides in medicinal crop ecosystems [19]. Biological insecticides are derived from a biological source that are intended for the elimination or control of insect pest. This biological insecticide mainly includes entomopathogenic fungi, bacteria, viruses and nematodes. Biorational pesticides refer to pesticides that are synthetic or natural substances that are effective against the target pest but have low or negligible toxicity to non-target organisms such as humans, animals, natural enemies and the environment [20]. These biorational pesticides includes microbial extracts, phytochemical (Plant-Incorporated protectants such as alkaloids, steroids, terpenoids, essential oils, phenolics) and biochemicals (hormones, botanicals, IGRs and semiochemicals) [21]. These insecticides of biological origin have a strong potential to replace the persistent conventional insecticides, can address ecological imbalance caused due to synthetic pesticides, and can ensure the food security. We hypothesized that evaluating the bioefficacy of biorational insecticides against *P. solenopsis* and *P. marginatus* on *G. sylvestre* will help in formulating eco-friendly pest management practices.

## 2 Materials and methods

### 2.1 Study site

The laboratory experiments were carried out in the Department of Entomology, ICAR-Directorate of Medicinal and Aromatic Plants Research (DMAPR), Anand, Gujarat, India (22° 35′56.5 N, 72° 55′ 60.0” E, 45.2 m above Mean Sea Level).

### 2.2 Experimental design

The experimental design used a Completely Randomized Design (CRD) with twelve treatments and three replications for each spray and each mealybug species.

### 2.3 Insect source

The incidence of *P. solenopsis* and *P. marginatus* were observed in *G. sylvestre* fields of ICAR- DMAPR (22° 35′50.8 N, 72° 56′ 04.8” E, 45.1 m above Mean Sea Level). The severity of the mealybug infestation was found to be more during the summer months (April to August). Insecticide-free colonies of mealybugs, which had not been treated with insecticides for a minimum of 30 days, were collected along with infested plant part/shoots. The plants parts containing the mealybugs were removed and placed in transparent zip lock plastic bags (12 × 6 Inches) and taken to the laboratory conditions for further observation and rearing. The collected insects were sent to Dr. Sunil Joshi, ICAR- National Bureau of Agricultural Insect Resources, Bengaluru for identification. The identified mealybug species were maintained and reared up to F2 generation at 26 ± 2 °C, 65 ± 5 % RH and 12:12 light: dark photoperiod. The culture's purity was maintained by using sprouted potatoes as a food source.

### 2.4 Rearing of mealybug species on potato sprouts

The initial culture of *P. solenopsis* and *P. marginatus* were reared on sprouted potatoes, *Solanum tuberosum* (L.), following the method suggested by Dhobi and Mehta [22] and Nabil [23] with minor modifications. Potatoes were thoroughly washed with distilled water and left under shade for 30 min to become dry. Uniform-sized potatoes were taken in a plastic tray (30 × 20 × 3.0 cm) with 3/4th substrate of cleaned soil media treated with carbendazim (Bavistin®) at 5 g/kg to avoid fungal infection to the tubers. Water was sprinkled on alternative days to maintain moisture in the soil media and encourage the sprouts’ growth. Potato sprouts were ready for mealybug inoculation after 25–30 days, with each potato having 4–6 buds with 8–10 cm of sprouts. The matured female mealybugs, along with ovisacs, were transferred to each potato sprout with the help of a Fisherbrand™ small round tip camel hair brush. The inoculated plastic trays were kept in acrylic cages (60 cm l × 40 cm w × 30 cm h) separately to avoid cross-contamination of species. The released mealybugs settled on sprouts and started laying eggs. Nymphs emerged from the ovisacs (first instar nymph) are commonly referred to as crawlers. These crawlers are active in early stages of its life cycle and upon hatching, it search for suitable place and start feeding. After completion of the F2 generation, matured female adults were carefully removed from the potato sprouts and used for bioassay.

### 2.5 Plant material collection and preparation of extracts

Plant materials of holy basil, *Ocimum sanctum* L. (Lamiaceae); kalmegh, *Andrographis paniculata* (Burm. F.) Wall. ex Nees. (Acanthaceae); and custard apple, *Annona squamosa* L. (Annonaceae) were harvested from the farm field and botanical garden maintained in ICAR- DMAPR, Anand. The respective plant materials were packed in separate transparent zip lock plastic bags (12 × 16 inches) and labelled properly. The materials were brought to the laboratory, and the leaves were removed, washed with water, and air-dried in the shade at room temperature (25 ± 3 °C) for 5 days. The selected plant leaves were ground into a coarse powder using a mixer grinder (Philips 240 V-50 Hz/600 W) and preserved. The aqueous extract of each plant was prepared by adding 50 g of leaf powder in 500 mL sterile distilled water in a 1000 mL Erlenmeyer flask, which was then boiled for 15 min over a low flame and later cooled. After cooling, the contents of the flasks were filtered with double filter paper and sterile filters to remove any impurities and made into a final stock solution [24,25]. The concentration of 5 % extract was prepared using sterile distilled water. The extracts were stored separately in sterile airtight reagent bottle (1000 ml Borosilicate glass screw cap wide mouth containers), to prevent exposure to sunlight and heat. The extracts were stored in refrigerated condition at 4 °C. Freshly produced extracts were used every time for the bioassay investigation.

### 2.6 Commercial products

The commercial insect growth regulator (Buprofezin 25 % SC), microbial-originated insecticide (Spinosad 45 % SC), and botanical insecticide (Karanja oil, *Pongamia pinnata* (L.)) and Azadirachtin 1 % EC (10000 ppm), and were obtained from authenticated pesticide retailers shop of Anand, Gujarat. These products were evaluated by applying at the recommended field doses. The chemical insecticide, Acetamiprid 20 % SP was used as a standard check, and distilled water as a control(Table 1).

**Table 1:** Experimental treatments.

Designation	Treatments description (Chemical name/Scientific name/EPF	Concentration (g or mL/L of water)	Trade name	Manufacturer
T_1_	Azadirachtin 1 % EC (10000 ppm)	5 mL/L	Neem-A-Life®	Gujarat Life sciences Pvt Ltd
T_2_	Karanja oil	5 mL/L	–	Nexus Bio Science Pvt. Ltd.
T_3_	Buprofezin 25 % SC	0.5 mL/L	Apple®	Danuka Agritech Pvt Ltd
T_4_	*Ocimum sanctum* leaf extract	50 mL/L	–	–
T_5_	*Andrographis paniculata* leaf extract	50 mL/L	–	–
T_6_	*Annona squamosa* leaf extract	50 mL/L	–	–
T_7_	*Beauveria bassiana* [2 × 10^9^ spores/ml]	5 mL/L	Green Beauveria®	Green life Biotech Laboratory
T_8_	*Lecanicillium muscarium* [2 × 10^9^ spores/ml]	5 mL/L	Green Meta®	Green life Biotech Laboratory
T_9_	*Metarhizium anisopliae* [2 × 10^9^ spores/ml]	5 mL/L	Green verticell®	Green life Biotech Laboratory
T_10_	Spinosad 45 % SC	0.3 mL/L	Tracer®	Dow Agro Sciences Pvt Ltd
T_11_	Acetamiprid 20 % SP (Standard check)	0.2 g/L	Tufan®	Aroxa Crop Science Pvt Ltd
T_12_	Distilled water spray (Control)	Pure water	–	–

### 2.7 Entomopathogenic fungi (EPF)

The bio-efficacy of three entomopathogenic fungi-based biopesticides, *viz*. *Beauveria bassiana* Balsamo (Vuillemin) (Hypocreales: Cordycipitaceae) (2 × 10^9^ spores/mL) (Green Beauveria®), *Metarhizium anisopliae* (Metsch.) Sorok (Ascomycota: Hypocreales) (2 × 10^9^ spores/mL) (Green Meta®) and *Lecanicillium muscarium* (*Verticillium lecanii*) (Zimm.) Zare & W. Gams (Hypocreales: Cordycipitaceae) (2 × 10^9^ spores/mL) (Green Verticell®) were evaluated in the present study. These EPF were cultured on Potato Dextrose Agar (PDA) medium in Petri dishes and incubated at 25 ± 1 °C, 70 ± 5 % RH for 7 days in dark condition to confirm their viability [26].

### 2.8 Laboratory bioassay

The laboratory bioassay experiment was conducted following the procedure described previously on *G. sylvestre* [12] with minor modifications. Approximately 10–15 cm long terminal portions of twigs/shoots (containing 4 to 5 leaves) of *G. sylvestre* were collected from untreated plots. These plots were separately maintained in organic production plot at DMAPR, Anand. Subsequently, they were thoroughly washed with sodium hypochlorite (NaOCl @ 1 %) for 30s, rinsed with distilled water, and air-dried before applying the treatments. Each healthy twig/shoot was kept in sterilized Petri dishes (15 × 16 × 2.5 cm), and the end of the twig was put in an eppendorf tube containing sucrose solution to maintain its turgidity. Mature females of *P. solenopsis* and *P. marginatus* were randomly collected from a synchronized colony and released separately (n = 20) in each twig using a soft hairbrush. The mealybugs were allowed to settle on the twig/shoot. Our previous study results showed that the leaf spray method was superior to the leaf dipping method for achieving a higher percent mortality when applying different biorational insecticides to manage *A. nerii* on *G. sylvestre* under laboratory condition [12]. The chosen formulations were dissolved in tap water at an appropriate concentration based on the recommendation of the Central Insecticide Board and Registration Committee (CIB&RC) and previous research conducted on different sucking pests [12,27,28]. The spraying was performed using a Lakeer 250 mL Plastic spray bottle hand-held sprayer, and each treatment was replicated thrice. To disperse the spores uniformly in the suspension, 0.02 % Tween 80 was added to the EPF solution. Mealybugs were considered dead, if their legs did not move after being repeatedly prodded with a fine brush and if their color changed from brown to black. For EPF, the immobilized mealybugs were recorded as dead by observing the mycelium over the body of mealybugs under Leica DM1000 compound microscope. Furthermore, cadavers of the insect species were surface sterilized using absolute alcohol and then placed on SDYA (Sabouraud Dextrose Yeast Agar) medium to facilitate fungal growth and for additional confirmation.

### 2.9 Data collection and statistical analysis

The data on mortality of exposed insect species were examined at regular time intervals, *viz*. 24, 48, 72, 96 and 120 h post-treatment. The percent mortality data of *P. solenopsis* and *P. marginatus* were corrected using Abbott's formula [29]. The mean difference among the treatments was tested by one-way analysis of variance (ANOVA) at 1 % and 5 % levels of significance by using Duncan's multiple range test (DMRT) [30]. The statistical analysis was performed through WASP (version 2.0) statistical package. The LT_50_ and LT_90_ values were determined by probit analysis using the log-probit method (POLO-PLUSver.2.0). Fit of the regression lines was verified using the **χ**^**2**^ test, and the LT_50_ and LT_90_ values of the treatments were compared using confidence intervals (95 %).

## 3 Results

The treatments significantly showed a high cumulative per cent mortality at laboratory condition.

### 3.1 Efficacy of biorational insecticides on mealybug species

**Fig. 1:**
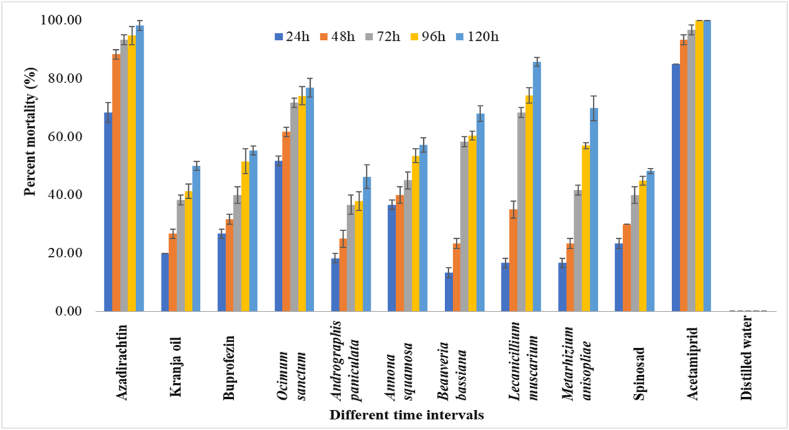
Percent mortality of biorational insecticides against *Phenacoccus solenopsis* on *Gymnema sylvestre* at different time intervals in first spray. The error bars represent the standard error of mean.

Different biorational insecticides applied at recommended doses significantly reduced the population of the mealybug species and showed greater efficiency in controlling *P. solenopsis* and *P. marginatus* in first spray. After 24 HAT, acetamiprid 20 % SP showed the highest mortality (85.00 %) compared to all other treatments, followed by azadirachtin 1 % EC (68.33 %) and *O. sanctum* leaf extract (51.67 %). Similarly, at 48 HAT, azadirachtin proved effective against both mealybugs, resulted in mortality of 88.33 % for *P. solenopsis* and 81.40 % for *P. marginatus*. On the other hand, the percent mortality rate for *P. solenopsis* caused by acetamiprid was 100 % at 96 HAT, but azadirachtin, *L. muscarium* and *O. sanctum* leaf extract exhibited a substantial level of percent mortality rate, respectively, of 94.73 %, 74.21 % and 74.12 %. The application of *L. muscarium* resulted in a cumulative percent mortality of 85.70 % in *P. solenopsis* at 120 HAT. Whereas, *M. anisopliae* and *B. bassiana* caused mortalities of 69.79 % and 67.93 % respectively in *P. solenopsis* at 120 HAT during the first spray (Fig. 1).

**Fig. 2:**
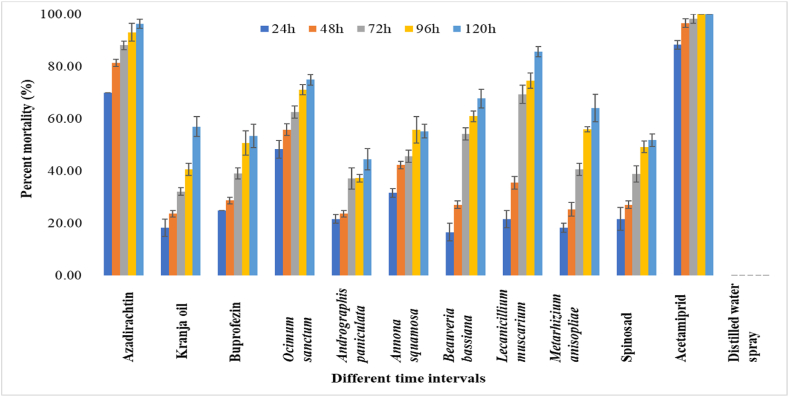
Percent mortality of biorational insecticides against *Paracoccus marginatus* on *Gymnema sylvestre* at different time intervals in first spray. The error bars represent the standard error of mean.

**Fig. 3:**
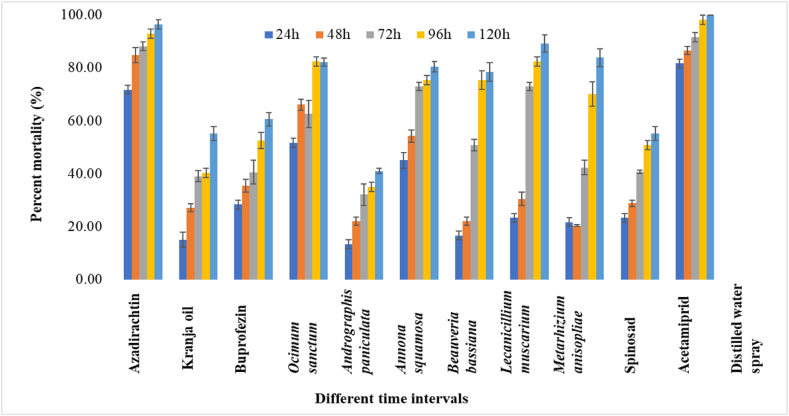
Percent mortality of biorational insecticides against *Phenacoccus solenopsis* on *Gymnema sylvestre* at different time intervals in second spray. The error bars represent the standard error of mean.

**Fig. 4:**
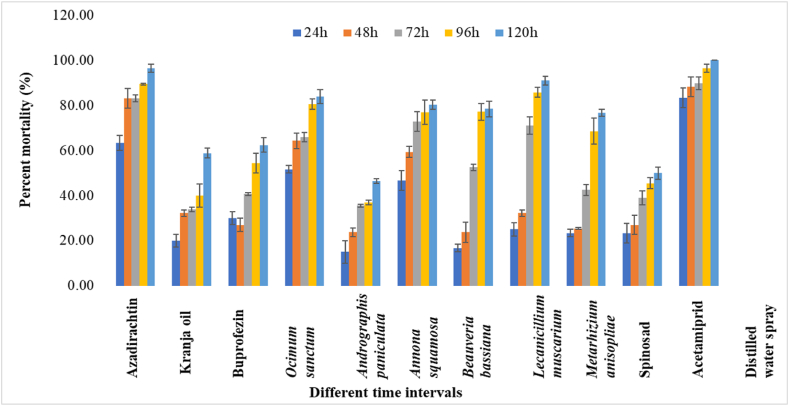
Percent mortality of biorational insecticides against *Paracoccus marginatus* on *Gymnema sylvestre* at different time intervals in second spray. The error bars represent the standard error of mean.

In *P. marginatus* 24 HAT, acetamiprid exhibited the highest population reduction with a percent mortality of 88.33 %, followed by azadirachtin and *O. sanctum* leaf extract, which resulted in mortalities of 70.00 and 48.33 %, respectively. On the other hand, azadirachtin and *L. muscarium* observed considerable mortality rate of 88.16 % and 69.38 %, respectively, at 72 HAT. After 96 HAT, 100 % mortality was recorded by acetamiprid, followed by azadirachtin (93.15 %). Meanwhile, after 120 HAT, azadirachtin caused a high population reduction, with a mortality of 96.39 %. *L. muscarium* and *O. sanctum* leaf extract showed 85.67 % and 74.90 %, mortality rate, respectively, against *P. marginatus* at 120 HAT. The mortality data for five time periods revealed the overall performance of all the biorational insecticides against the two mealybug species tested. It was observed that azadirachtin, *L. muscarium* and *O. sanctum* leaf extract were effective in causing mortality in both *P. solenopsis* and *P. marginatus*. However, *A*. *paniculata* leaf extract showed the least percent mortality of 46.29 % and 44.54 % against *P. solenopsis* and *P. marginatus*, respectively at 120 HAT (Fig. 2). In second spray, the percent mortality trends were more or less similar compared to first spray against *P. solenopsis* and *P. marginatus* (Fig. 3, Fig. 4) The overall pathogenicity trend of EPFs used in the study was *L. muscarium* > *M. anisopliae* > *B. bassiana* at 120h of post-treatment in both years.

### 3.2 Time-mortality response against biorational insecticides

The lethal effects of biorational insecticides against *P. solenopsis* and *P. marginatus* are documented in (Table 2, Table 3). Azadirachtin showed the lowest lethal time (LT_50_) of 12.52 h, while *A. paniculata* recorded the highest LT_50_ value (143.90 h) in *P. solenopsis*. Among the plant extracts, the extract of *O. sanctum* showed the lowest LT_50_ value of 24.50 h and among the EPF, *L. muscarium* showed the least LT_50_ value (52.20 h) in *P*. *solenopsis*. In *P. marginatus*, the lethal time values of azadirachtin, *O. sanctum* and *L. muscarium* were more or less similar. Overall, azadirachtin caused the highest percent mortality and least LT_50_ values, which were statistically significantly different from other biorational insecticides in both species. Since the standard check of acetamiprid resulted in more than 80 % mortality at the lowest exposure period, it was not possible to calculate the LT_50_ values for the same.

**Table 2:** Time-mortality response (LT_50_) (in hours) of different biorational insecticides against *Phenacoccus solenopsis*

Insecticide/bioagent	df^a^	Slope ± SE	χ^2^	LT_50_ (95 % FL) (hours)
Azadirachtin 1 % EC (10000 ppm)	3	1.846 ± 0.276	0.740	12.521 (6.856–17.686)
Karanja oil	3	1.498 ± 0.230	1.491	109.983 (91.266–146.329)
Buprofezin 25 % SC	3	1.257 ± 0.218	3.392	85.897 (62.067–160.655)
*Ocimum sanctum*	3	1.210 ± 0.218	1.970	24.520 (14.269–32.795)
*Andrographis paniculata*	3	1.342 ± 0.233	0.611	143.905 (111.916–225.885)
*Annona squamosa*	3	1.481 ± 0.216	0.054	51.124 (42.035–60.026)
*Beauveria bassiana* [2 × 10^9^ spores/mL]	3	2.724 ± 0.246	9.039	68.061 (50.844–92.297)
*Lecanicillium muscarium* [2 × 10^9^ spores/mL]	3	3.052 ± 0.246	10.797	52.202 (36.178–68.000)
*Metarhizium anisopliae* [2 × 10^9^ spores/mL]	3	0.019 ± 0.002	3.955	79.141 (68.258–91.379)
Spinosad	3	1.277 ± 0.221	1.422	102.221 (83.365–140.874)

a Degrees of freedom, LT (lethal time).

**Table 3:** Time-mortality response (LT_50_) (in hours) of different biorational insecticides against *Paracoccus marginatus*

Insecticide/bioagent	df^a^	Slope ± SE	χ^2^	LT_50_ (95 % FL) (hours)
Azadirachtin 1 % EC (10000 ppm)	3	1.701 ± 0.257	2.337	13.872 (7.681–19.447)
Karanja oil	3	1.488 ± 0.228	6.283	107.848 (73.226–416.862)
Buprofezin 25 % SC	3	0.010 ± 0.002	1.652	91.890 (81.356–106.219)
*Ocimum sanctum*	3	1.207 ± 0.217	2.261	26.583 (16.070–35.001)
*Andrographis paniculata*	3	1.251 ± 0.229	1.526	140.219 (108.096–226.483)
*Annona squamosa*	3	1.175 ± 0.213	0.172	42.305 (30.780–52.166)
*Beauveria bassiana* [2 × 10^9^ spores/mL]	3	2.583 ± 0.241	6.921	66.278 (50.927–86.510)
*Lecanicillium muscarium* [2 × 10^9^ spores/mL]	3	2.932 ± 0.242	12.032	49.828 (32.165–66.540)
*Metarhizium anisopliae* [2 × 10^9^ spores/mL]	3	2.117 ± 0.232	10.713	73.329 (49.128–129.380)
Spinosad	3	1.299 ± 0.223	1.768	106.492 (86.653–148.039)

a Degrees of freedom, LT (lethal time).

## 4 Discussion

Biorational insecticides are a long-term approach over synthetic pesticides to control various insects [31]. Biopesticides are important for sustainable agriculture because they are safe for soil and environment, have less toxicity to non-target organisms and are biodegradable [32]. The present study recorded that azadirachtin 10000 ppm is effective against *P. solenopsis* and *P. marginatus*. These findings align with the results reported by Saicharan et al. [27] who found that azadirachtin 10,000 ppm (64.49 %) effectively managed sucking pest like Chrysanthemum aphid*, Macrosiphoniella sanbornii* (Gillette), (Hemiptera: Aphididae). Further, Shivakumara et al. [12] reported 100% mortality in *Aphis ner*ii (Boyer de Fonscolombe), (Hemiptera: Aphididae) at 96 h after treatment with Azadirachtin 1 % under laboratory conditions at 25 ± 1 °C, 70 % ± 5 % RH. Our results showed that *O. sanctum* plant extracts significantly reduced the population of the both mealybug species. Lamiaceae family has been thoroughly documented as a potential insecticide for various pests. Clemente et al. [33] reported that *O. sanctum* exhibited the highest insecticidal action, resulting in significant control of cotton mealybug compared to extracts from different plants at lower concentrations. Bala et al. [34] observed 100 % repellency in methanol leaf extracts of *A. paniculata* (10 %) against *P. solenopsis*. However, our study showed less mortality and a high LT_50_ value in *A. paniculata* plant extract against both mealybug species. Methanol, a polar solvent, exhibits the highest bioactive compound and provides a better extractive yield in botanical pesticides when compared to aqueous solutions. Methanol extracts are more toxic than aqueous, and the type of extraction solvent used greatly affects the biological effects of biological compounds. The present study used an aqueous solution for extraction; therefore, the least mortality was recorded compared to the methanol leaf extract of *A. paniculata*. The biological activity is rationalized regarding the polarity of the compounds extracted by solvent. Thus, compared to aqueous, methanol can dissolve organic compounds more quickly, so the compound methanol extracts are more toxic and have biological activity. *Annona squamosa* plant extract, commonly known as custard apple, had shown potential for pest control across an extensive array of insect pests [35]. Our results showed that application of *A. squamosa* resulted in a respective mortality of 57.21 % against *P. solenopsis* and 52.22 % against *P. marginatus*. Buprofezin treatment caused mortality of 55.36 % and 53.40 % in *P. solenopsis* and *P. marginatus* respectively during the first spray. Buprofezin is a chitin biosynthesis inhibitor which interferes with cuticle formation and is the most widely applied IGR against mealybugs [36,37]. Muthukrishnan et al. [38] observed that buprofezin reduced the nymphal and adult populations of the pink hibiscus mealybug, *Maconellicoccus hirsutus* (Green) (Hemiptera: Pseudococcidae). Shoma et al. [39] observed that buprofezin recorded cent per cent mortality in *P. marginatus* populations.

Entomopathogenic fungi are viable alternatives to chemical insecticides for integrated pest management [40]. About 750 species of EPF in 90 genera can infect insect pests and mites [41]. Among them only a few EPF like *B. bassiana, M. anisopliae* and *L. muscarium* were evaluated against mealybug species [[42], [43], [44], [45], [46], [47]]. Unlike viruses and bacteria, EPF do not have to be ingested by an insect, they can directly penetrate the cuticle of the insets [48]. The infection process begins with the spore's production by two stages: the first stage relies on the action of hydrophobic and electrostatic forces and while the second step requires the activity of enzymes and low-molecular-weight proteins termed hydrophobins [49]. At the right temperature and humidity, the spore germination will start on the insect cuticle [49]. As appressoria develop, the cuticle is subjected to intense mechanical pressure and lytic enzymes are produced, which break down the cuticle [50]. The fungus hyphae begin to proliferate after they have entered the insects body cavity. The hosts physiological functions, particularly its immunological responses, are disrupted at this stage by the secondary metabolites produced by fungus [51]. The insect will eventually die as a result of the growing infection, which causes mechanical damage to the internal organs caused by growing hyphae and nutrition depletion [52]. Our study shows that *L. muscarium* (2 × 10^9^ spores/mL) at 5 g/l showed the highest per cent mortality in both mealybug species when compared to *B. bassiana* and *M. anisopliae*. The findings were confirmed by Banu et al. [28], who observed the highest virulence of *V. lecanii* against *P. solenopsis* and *P. marginatus.* Application of L. *lecanii* resulted in highest mortality of (73.33 %) mealybugs at 144 h after treatment, followed by *M. anisopliae* (63.33 %) and *B. bassiana* (56.66 %), respectively. Furthermore, *L. muscarium* exhibited a higher mortality rate than *B. bassiana* against the grape mealybug [21].

The LT_50_ value of Azadirachtin 1 % EC against *P. solenopsis* and *P. marginatus* were 12.52 and 13.87 h, respectively. The current findings were supported by Aljedani et al. [53], who recorded LT_50_ value of azadirachtin 1 % EC which takes nearly 28.30 h to cause 50 % mortality against the black watermelon bug, *Coridus viduatus* (Fabricius, 1794) (Hemiptera: Dinidoridae). The entomopathogenic fungus (EPF), *L. muscarium*, in our study exhibited significantly longer LT_50_ values, *i.e.* 52.20 h and 49.82 h in *P. solenopsis* and *P. marginatus,* respectively. The present findings were supported by Amala et al. [43], who found that mealybugs treated with *B. bassiana* and *M. anisopliae* had LT_50_ values of 3.04 days and 7.05 days, respectively, while mealybugs treated with *V. lecanii* had a substantially lower LT_50_ value of 1.09 days in *M. hirsutus*. Besides, *V. lecanii* showed the highest mortality (72.20 %) over *B. bassiana* and *M. anisopliae* in grape mealybug [43]. Amutha and Banu [54] recorded LT_50_ values of *M. anisopliae* as 5.66–6.27 days and 6.52–7.02 days against *P. solenopsis* and *P. marginatus* respectively.

## 5 Conclusion

The results obtained from the present study showed that the biorational insecticide, specifically Azadirachtin 1 % EC (10000 ppm), resulted in a higher percentage of mortality and the shortest median lethal time among all the tested biorational insecticides for both mealybug species. Hence, the proven biopesticides can be included in the integrated pest management program against *P. solenopsis* and *P. marginatus* in the *G. sylvestre* ecosystem. Besides, *O. sanctum* leaf extract and EPF, *L. muscarium* (2 × 10^9^ spores/mL), *M. anisopliae* (2 × 10^9^ spores/mL), and *B. bassiana* (2 × 10^9^ spores/mL) also caused high mortality against *P. solenopsis* and *P. marginatus*. Among the EPF, *L. muscarium* showed the highest virulent nature with the lowest LT_50_ value in both species.

## Funding

Researchers supporting project (RSPD2024R741), 10.13039/501100002383King Saud University for publication of this manuscript.

## Ethical approval

This article does not contain any studies with human participants or animals.

## Data availability statement

Data included in article/supplementary material/referenced in article.

## Additional information

No additional information is available for this paper.

## CRediT authorship contribution statement

**Shivakumara Kadanakuppe Thammayya:** Conceptualization, Investigation, Writing - original draft. **Keerthi Manikyanahalli Chandrashekara:** Data curation, Writing - review & editing. **Akula Chinapolaiah:** Data curation, Formal analysis. **Ramya Ramesan Syamala:** Writing - review & editing. **Shivakumar Kadukothanahalli Veerabhadraiah:** Data curation, Formal analysis. **Bhemanna Somanna Gotyal:** Methodology, Writing - review & editing. **Manjunatha Channappa:** Formal analysis, Methodology. **Ryan Casini:** Supervision. **Ihab Mohamed Moussa:** Writing - review & editing. **Hosam O. Elansary:** Funding acquisition, Supervision. **Ahmed M. El-Sabrout:** Supervision.

## Declaration of competing interest

The authors declare that they have no conflict of interest.
